# Perioperative, functional, and oncologic outcomes of laparoscopic partial nephrectomy versus open partial nephrectomy for complex renal tumors: a systematic review and meta-analysis

**DOI:** 10.3389/fonc.2023.1283935

**Published:** 2024-01-10

**Authors:** Fan Zhang, Jiang-sheng Hu, Kai-yu Zhang, Xiao-hua Liu

**Affiliations:** Department of Urology, Minda Hospital of Hubei Minzu University, En Shi, China

**Keywords:** laparoscopic partial nephrectomy, open partial nephrectomy, complex renal tumors, meta-analysis, outcomes

## Abstract

**Background:**

The primary aim of this present study is to undertake a comprehensive comparative analysis of the perioperative, functional, and oncologic outcomes associated with laparoscopic partial nephrectomy (LPN) and open partial nephrectomy (OPN) as interventions for the treatment of complex renal tumors, defined as PADUA or RENAL score ≥ 7.

**Methods:**

We systematically carried out an extensive search across four electronic databases, namely PubMed, the Cochrane Library, Embase, and Web of Science. Our objective was to identify pertinent studies published in the English language up to December 2023, and encompassed controlled trials comparing LPN and OPN as interventions for complex renal tumors.

**Results:**

This study encompassed a total of seven comparative trials, involving 934 patients. LPN exhibited a noteworthy reduction in the length of hospital stay (weighted mean difference [WMD] -2.06 days, 95% confidence interval [CI] -2.62, -1.50; p < 0.00001), blood loss (WMD -34.05mL, 95% CI -55.61, -12.48; p = 0.002), and overall complications (OR 0.38, 95% CI 0.19, 0.79; p = 0.009). However, noteworthy distinctions did not arise between LPN and OPN concerning parameters such as warm ischemia time, renal function, and oncological outcomes.

**Conclusions:**

This study reveals that LPN presents several advantages over OPN. These benefits encompass a shortened hospital stay, diminished blood loss, and a reduced incidence of complications. Importantly, LPN achieves these benefits while concurrently upholding comparable renal function and oncological outcomes.

**Systematic Review Registration:**

https://www.crd.york.ac.uk/prospero/display_record.php?RecordID=457716, identifier CRD42023453816.

## 1 Introduction

Renal tumors constitute a prevalent form of urinary system neoplasm, ranking second in incidence only to bladder cancer. Owing to the inherently low biological aggressiveness of renal tumors, their malignancy is relatively subdued (1). In recent years, the continuous advancements in medical diagnostic technology have contributed to heightened rates of early kidney tumor detection and favorable clinical prognoses. Clinical investigations affirm the inherent resistance of renal tumors to multiple pharmaceutical agents and their reduced sensitivity to radiation, thereby constraining options for biological targeting and immunotherapy (2). Consequently, surgical intervention has emerged as the foremost efficacious modality for managing renal tumors. Nephron-sparing surgery represents a surgical approach endorsed in the clinical treatment paradigm for renal tumors. This technique adeptly conserves the patient’s nephron while concurrently extending their survival duration to a significant degree (3, 4).

RENAL and PADUA score rank among the most frequently employed scoring systems in the assessment of renal tumors. Complex renal tumors are defined by a PADUA or RENAL score of ≥ 7 (5, 6). Owing to the deep encapsulation of the renal parenchyma and the intricate proximity to the anatomically elaborate renal collection system, the challenge of excising or resecting complex renal tumors while preserving nephrons is evident. Laparoscopic partial nephrectomy (LPN) has gained broad acceptance across numerous medical institutions due to its straightforward requirements for surgical equipment. Its effectiveness has been validated in managing non-complex renal tumors (7). Nonetheless, the utilization of LPN for the management of complex renal masses continues to be a subject of ongoing debate and intricacy (8). Despite numerous recent endeavors to scrutinize and compare the perioperative and functional outcomes of LPN and open partial nephrectomy (OPN) in addressing complex renal tumors, a substantial portion of these investigations remains constrained within the boundaries of single medical institutions or the realm of proficient surgical practitioners (9).

Hence, the primary objective of this investigation is to systematically aggregate data from comparative studies and conduct a comprehensive assessment of the effectiveness and safety profiles pertaining to LPN and OPN in the management of exceedingly intricate renal tumors. The outcomes derived from this inquiry are intended to serve as all-encompassing guide for clinical deliberations, thereby aiding medical practitioners in the judicious determination of the optimal surgical modality for their respective patient cohorts.

## 2 Methods

This study was conducted in strict adherence to the protocols outlined in the Preferred Reporting Items for Systematic Reviews and Meta-Analyses (PRISMA) statement (10, 11). Moreover, it was registered in the PROSPERO registry (ID: CRD42023457716) in accordance with established practices.

### 2.1 Literature search strategy, study selection and data collection

A rigorous and exhaustive exploration was conducted across a multitude of databases, encompassing PubMed, Embase, Web of Science, and the Cochrane Library. Our objective was to identify pertinent studies published in the English language up to December 2023. We formulated the subsequent search query through the amalgamation of intervention and patient-centric search phrases: ((Laparoscopic PN OR Endoscopic PN) AND (Renal carcinoma OR Renal tumor OR Renal cancer OR Kidney cancer) AND (Complex)). In order to ascertain comprehensiveness, we also carried out a manual examination of pertinent references, proceedings, and summaries.

We used the PICOS approach to define inclusion criteria. P (patients): adult patients diagnosed with complex renal tumor, defined specifically by a PADUA or RENAL score of ≥ 7; I (intervention): the intervention involves patients undergoing LPN; C (comparator): OPN was performed for comparison; O (outcome): one or more of the ensuing outcomes: perioperative, complications, renal functional, and oncologic outcomes; S (study type): This investigation encompasses prospective comparative studies, retrospective analyses, or randomized controlled trials (RCTs). Exclusion criteria were as follows: (1) studies lacking comparative designs were systematically excluded. (2) editorial commentaries, correspondences with the editor, abstracts from meetings, and isolated case reports were not incorporated into the analytical framework. (3) studies that did not assess the designated outcome metrics were deliberately excluded from consideration.

Subsequently, two independent reviewers meticulously extracted the following dataset from the included studies: (1) General manuscript details, including the year of publication, author, and country of origin. (2) Attributes of the study cohort, including sample size, age distribution, and body mass index (BMI). (3) Tumor site, preoperative estimated glomerular filtration rate (eGFR), RENAL score, and follow-up duration. (4) Perioperative consequences: Duration of the operation, volume of blood loss, instances of transfusion, length of hospital stay, warm ischemia, conversion to radical nephrectomy, positive surgical margins (PSM), overall complications (defined by Clavien grade ≥ 1), and major complications (defined by Clavien grade ≥ 3) (12). The process of data extraction was autonomously conducted by the two reviewers to ensure meticulousness and consistency.

In order to appraise the quality of the literature, a comprehensive evaluation was undertaken on the studies incorporated in the analysis, employing the “risk of bias in non-randomized studies of interventions” (ROBINS-I) framework (13). This assessment was independently conducted by two evaluators (F.Z. and J.H.), who scrupulously scrutinized the studies for potential biases, encompassing confounding variables or other plausible origins of systematic divergence. Any incongruities or disparities that emerged during the assessment procedure were resolved through thorough deliberations.

### 2.2 Statistical analysis

For the purpose of data analysis in this study, we used the Cochrane Collaborative RevMan 5.4 software. Odds ratios (OR) were employed to assess dichotomous outcomes, while weighted mean differences (WMD) were used to quantify continuous outcomes, accompanied by 95% confidence intervals (CI) for all measured parameters. The evaluation of inter-study heterogeneity was conducted using the I^2^ test (14). In anticipation of potential inter-trial heterogeneity, we adopted the random-effects model for all analytical procedures, establishing a level of statistical significance at p < 0.05. In scenarios where substantial heterogeneity was identified among outcomes (I^2^ > 75%), sensitivity analyses were executed to pinpoint the origins of inter-study variance and to authenticate the steadfastness of our conclusions. It’s noteworthy, however, that sensitivity analyses were precluded for outcomes derived from three or fewer studies.

### 2.3 Publication bias

To appraise the likelihood of publication bias, we employed Begg’s method funnel plot.

## 3 Results

### 3.1 Baseline characteristics

**Figure 1:**
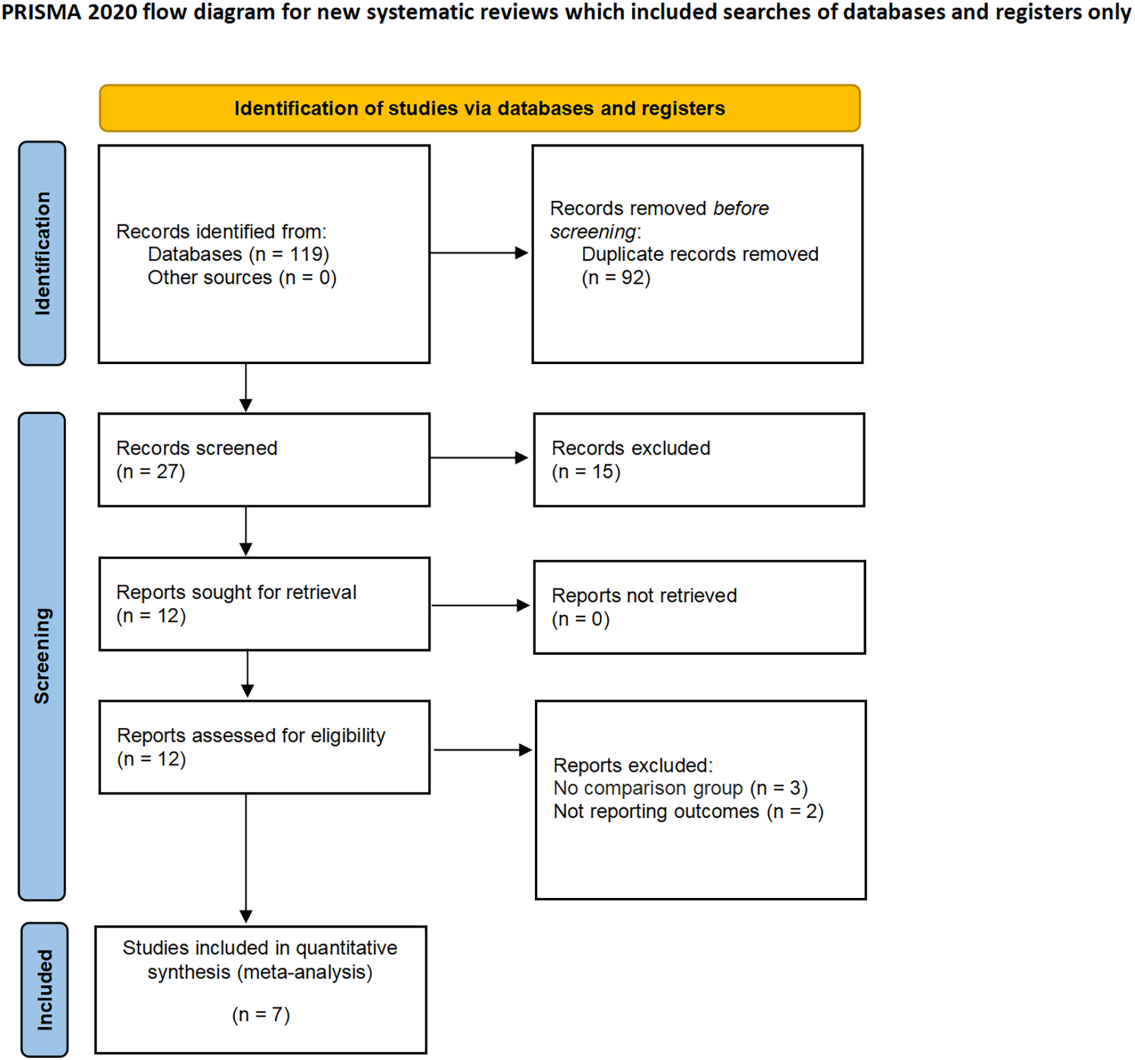
PRISMA flow diagram for the systematic review.

The applied search algorithm initially identified a total of 116 studies within the databases. Following an extensive review of full-text materials and a meticulous screening process, seven studies, comprising 934 patients in total (432 LPN vs. 502 OPN), were deemed suitable for inclusion in the comprehensive meta-analysis (**Figure 1**) (15–21). The succinct overview in **Table 1** offers a synopsis of fundamental patient characteristics, accompanied by the corresponding interventions and associated preoperative variables (including sample size, age, BMI, tumor diameter, preoperative eGFR, RENAL score, and follow-up duration). These studies were published between 2021 and 2023. **Table 2** delineates perioperative and surgical outcomes, encompassing pivotal parameters such as operative temporal metrics, blood loss, instances of transfusion, duration of hospitalization, warm ischemic intervals, cases necessitating conversion to the more extensive radical nephrectomy, enucleation, PSM, and complications. The renal functional and oncologic outcomes is presented in **Table 3**.

**Table 1:** The trials included in the systemic review.

Reference	Year	Country	Design of study	Age(y)		BMI (kg/m2)		Patients		Tumor site (Lt/Rt)		Preoperative eGFR (ml/min/1.73 m)		Score used	Follow-up duration
				LPN	OPN	LPN	OPN	LPN	OPN	LPN	OPN	LPN	OPN		
Giulioni (15)	2023	Italy	Retrospective	64(13)	64(12)	26.9(3.9)	26.1(2.4)	76	137	39/37	70/67	94.1(34.6)	94.5(33.6)	RENAL score≧̸7	LPN: 41(22) months; OPN: 45(24) months
Liu (16)	2022	China	Retrospective	57(9.26)	54.5(8.52)	24.5(3.19)	26.10(5.41)	97	44	60/37	25/19	100(24.78)	92.07(30.69)	PADUA score≧̸10	54 months
Guo (17)	2021	China	Retrospective	52.31(4.61)	53.18(4.52)	21.15(1.03)	21.23(0.96)	24	26	13/11	12/4	affected side: 43.37(6.83)	affected side: 43.42(6.91)	RENAL score>7	3 years
Li (18)	2021	China	Retrospective	57.6(11.5)	58.5(12.15)	NA		20	20	11/9	10/10	NA		RENAL score>9	NA
Yu (19)	2021	China	Retrospective	52.16(8.43)	53.46(8.51)	24.6(3.1)	25(2.9)	66	66	32/34	33/33	75.12(10.24)	75.86(10.36)	RENAL score>7	NA
Chiancone (20)	2021	Italy	Retrospective	60.15(10.39)	59.19(10.6)	26.3(1.9)	26.8(1.5)	72	21	38/34	11/10	NA		PADUA score≧̸10	NA
Mari (21)	2021	Italy	Prospective	63.9(13.19)	64.1(13.19)	26.1(3.33)	25.5(3.7)	77	188	NA		81.9(26)	83.7(20.37)	PADUA score≧̸10	48 months

LPN, laparoscopic partial nephrectomy; OPN, open partial nephrectomy; RPN, robotic partial nephrectomy; eGFR, estimated glomerular filtration rate; CCI, charlson comorbidity index; Mean (SD).

**Table 2:** Surgical outcomes.

Reference	Operative time(mins)		Blood loss (ml)		Transfusion (n)		Length of stay (days)		Warm ischemia time (mins)		conversion to radical nephrectomy (n)		Enucleation (n)		PSM (n)		Major complications (n)		Overall complications (n)	
	LPN	OPN	LPN	OPN	LPN	OPN	LPN	OPN	LPN	OPN	LPN	OPN	LPN	OPN	LPN	OPN	LPN	OPN	LPN	OPN
Giulioni (15)	132(49)	144(52)	249(114)	329(269)	3	9	4.7(1.9)	6.1(2.3)	0	7.5(10.37)	2	1	NA		3	8	3	5	17	30
Liu (16)	156.00(47.78)	160.00(48.15)	150.00(74.07)	200(127.32)	5	0	7(2.96)	10(4.44)	30.69(5.45)	19.93(3.41)	0	0	NA		3	0	2	2	7	6
Guo (17)	102.35(8.92)	95.61(9.23)	100.25(10.72)	126.71(9.84)	NA		8.24(1.47)	10.18(1.34)	18.94(4.52)	23.41(4.61)	NA		NA		NA		NA		1	7
Li (18)	135.1(12.2)	121.9(10.2)	162.3(30.6)	125.2(45.5)	NA		10.2(2.1)	12.8(1.5)	23.1(5.2)	15.2(4.3)	NA		NA		NA		NA		0	0
Yu (19)	NA		152.48(20.16)	201.75(23.95)	NA		10.74(2.15)	14.46(3.87)	28.15(6.86)	21.27(4.29)	0	0	NA		NA		NA		2	10
Chiancone (20)	143.06(48.71)	146.43(48.9)	309.72(152.67)	407.14(208.73)	3	7	6.39(1.03)	7.33(1.02)	NA	6.5	1	1	68	18	3	2	3	2	11	12
Mari (21)	148(77.78)	138(54.07)	150(148.15)	200(222.2)	6	26	3(0.74)	5(0.74)	18(7.41)	19(5.93)	NA		23	22	15	13	7	18	13	47

LPN, laparoscopic partial nephrectomy; OPN, open partial nephrectomy; PSM, positive surgical margins; Mean (SD).

**Table 3:** Renal functional and oncologic outcomes.

Reference	eGFR decline (ml/min/1.73 m)		Local recurrence (n)		Tumor size (cm)		Pathological stage (pT) OR Clinical stage (cT)	
	LPN	OPN	LPN	OPN	LPN	OPN	LPN	OPN
Giulioni (15)	5.9(32.5)	7.1(33.2)	NA		NA		pT1a:17; pT1b:43; pT2:16	pT1a:58; pT1b:55; pT2:24
Liu (16)	18.78(15.77)	2.57(6)	NA		NA		pT1b:94; pT2a:3; pT2b:0	pT1b:41; pT2a:2; pT2b:1
Guo (17)	5.11(6.12)	10.28(6.2)	NA		3.16(0.53)	3.23(0.48)	cT1a:16; cT1b:8	cT1a:19; cT1b:7
Li (18)	NA		NA		6.8(1.6)	6.6(1.9)	All are cT1 and cT2	
Yu (19)	11(9.88)	9.42(9.99)	NA		2.76(0.86)	2.8(0.76)	All are cT1 and cT2	
Chiancone (20)	NA		NA		NA		cT1a:5; cT1b:14; cT2a:2	cT1a:16; cT1b:49; cT2a:7
Mari (21)	10.7(12.3)	13.2(11.93)	NA		<4: 36; 4-7:34; ≥7:7	<4: 96; 4-7:73; ≥7:19	pT1a:26; pT1b:33; pT2:2; pT3a:8	pT1a:79; pT1b:52; pT2:3; pT3a:18

LPN, laparoscopic partial nephrectomy; OPN, open partial nephrectomy; eGFR, estimated glomerular filtration rate; Mean (SD).

No significant differences were observed in terms of age (p = 0.69), laterality (p = 0.66), BMI (p = 0.08), and preoperative eGFR (p = 0.86) between the LPN and OPN groups (**Table 4**).

**Table 4:** Comparison of baseline patient.

Baseline characteristic	LPN VS OPN	Heterogeneity I^2^ (%)	*p* value
Age WMD (95% CI)	-0.05(-1.35 to 1.24)	0	0.69
Left side OR (95% CI)	1.07(0.78 to 1.48)	0	0.66
BMI WMD (95% CI)	-0.06(-0.58 to 0.45)	49	0.08
Preoperative eGFR WMD (95% CI)	-0.26(-3.09 to 2.57)	0	0.86

LPN, laparoscopic partial nephrectomy; OPN, open partial nephrectomy; eGFR, estimated glomerular filtration rate.

### 3.2 Assessment of quality

All of the studies conducted a comparative analysis and were published between 2021 and 2023. Out of these, three studies were found to exhibit a moderate level of bias risk, while four studies demonstrated a significantly high risk of bias (**Supplementary Table 1**).

### 3.3 Outcome analysis

#### 3.3.1 Perioperative effectiveness

**Figure 2:**
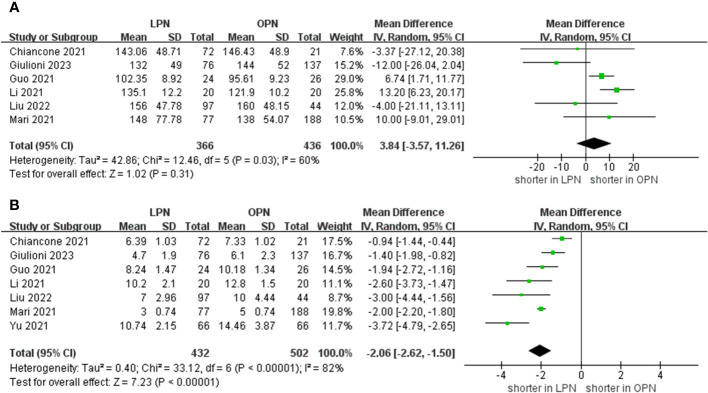
Forest plots of perioperative outcomes **(A)** operative time, **(B)** length of hospital stay.

The meta-analysis encompassed six studies that provided data on operative time. Additionally, the results demonstrated that there was no statistically significant distinction in operative time between the groups that underwent LPN and OPN (WMD 3.84 mins, 95% CI -3.57 to 11.26; p = 0.31) (15–18, 20, 21). After pooling data from seven separate studies, the LPN cohort demonstrated a shorter duration of hospitalization in comparison to their OPN counterparts (WMD -2.06 days, 95% CI -2.62 to -1.50; p < 0.00001) (**Figure 2**) (15–21).

**Figure 3:**
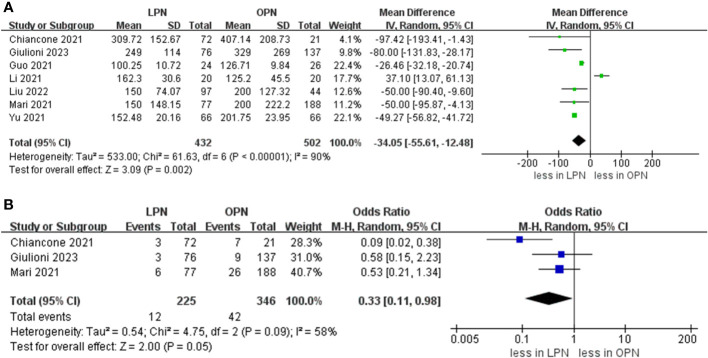
Forest plots of perioperative outcomes **(A)** blood loss, **(B)** transfusion rates.

**Figure 4:**
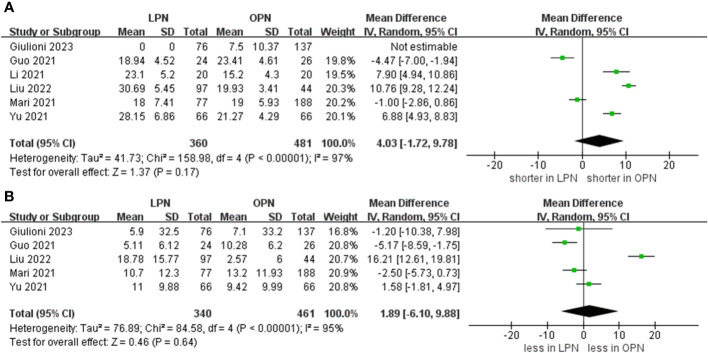
Forest plots of perioperative and renal functional outcomes **(A)** warm ischemia time, **(B)** eGFR decline.

Data concerning blood loss were extracted from seven studies (15–21). The collective findings indicated that LPN was linked to lower blood loss compared to OPN (WMD: -34.05 mL, 95% CI: -55.61 to -12.48; p = 0.002). Nevertheless, no significant distinction was observed in the transfusion rate prevalence between LPN and OPN (OR 0.33, 95% CI 0.11, 0.98; p = 0.05) (three studies; **Figure 3**) (15, 20, 21). The analysis revealed that a discernible disparity in the prevalence of warm ischemia time between LPN and OPN was not evident (WMD 4.03 mins, 95% CI -1.72 to 9.78; p = 0.17) (based on findings from six studies; **Figure 4**) (15–19, 21).

#### 3.3.2 Renal functional

The meta-analysis encompassed four studies that reported the rate of decline in eGFR. The results demonstrated that there was no statistically significant distinction in the eGFR decline between the groups that underwent LPN and OPN (WMD 1.89 ml/min/1.73 m, 95% CI -6.10 to 9.88; p = 0.64) (**Figure 4**) (15–17, 19, 21).

#### 3.3.3 Complications

**Figure 5:**
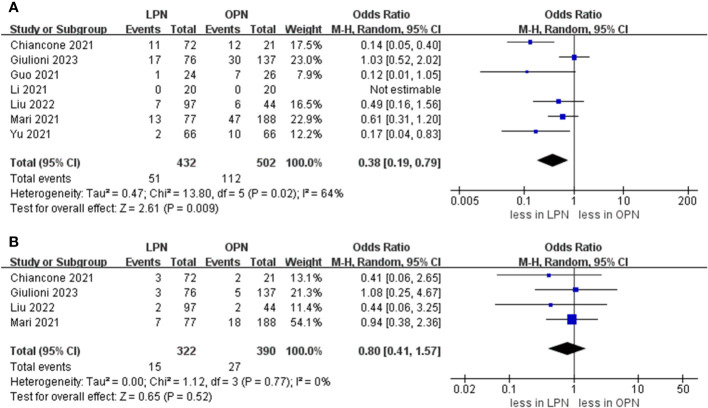
Forest plots of complication **(A)** overall complications, **(B)** major complication.

The cumulative incidence of overall complications was 11.8% (51 out of 432 cases) for LPN and 22.3% (112 out of 502 cases) for OPN. Across seven studies (16–21), LPN exhibited a lower incidence of overall complications in comparison to OPN (OR 0.38, 95% CI 0.19, 0.79; p = 0.009) (15–21). Furthermore, the rates of major complications were 4.5% (15 out of 332 cases) for LPN and 6.9% (27 out of 390 cases) for OPN. However, no statistically noteworthy disparities in the occurrence of significant complications between LPN and OPN were detected (OR 0.80, 95% CI 0.41, 1.57; p = 0.52) (**Figure 5**) (15, 16, 20, 21).

#### 3.3.4 Oncologic outcomes

**Figure 6:**
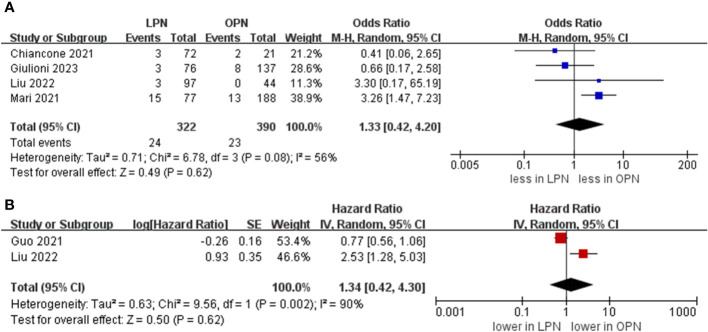
Forest plots of oncologic outcomes **(A)** PSM, **(B)** overall survival (5 years).

A PSM-based meta-analysis demonstrated that there existed no statistically meaningful distinction between LPN and OPN (four studies; OR 0.80, 95% CI 0.41, 1.57; p = 0.52) (15, 16, 20, 21). Moreover, no statistically notable differentiation was witnessed regarding overall survival (OS) (5 years) between LPN and OPN (two studies; HR 1.34, 95% CI 0.42, 4.30; p = 0.62) (**Figure 6**) (16, 17).

### 3.4 Heterogeneity

A prevailing inclination toward mild to moderate heterogeneity was noticeable across the majority of the outcomes. Although studies of intermediate and high quality were incorporated, substantial heterogeneity emerged in four of the outcomes: duration of hospitalization (I^2 =^ 82%), blood loss (I^2 =^ 90%), warm ischemia time (I^2 =^ 97%), and eGFR decline (I^2 =^ 95%).

### 3.5 Sensitivity analysis

In this study, the identification of conspicuous heterogeneity in aspects such as duration of hospital stay, blood loss, warm ischemia time, and eGFR decline prompted the implementation of a sensitivity analysis. The aim was to precisely determine the fundamental origin of this heterogeneity and to assess the robustness and coherence of the study’s conclusions. The outcomes derived from this analysis revealed the absence of noteworthy fluctuations in the extent of heterogeneity, thus implying a consistent source of heterogeneity in terms of the duration of hospital stay, blood loss, warm ischemia time, and eGFR decline (as depicted in **Supplementary Table 2**).

### 3.6 Publication bias

To evaluate the possible existence of publication bias in the examined research, we performed an analysis centered on variables including operative time, length of hospital stay, warm ischemia time, and overall complications. Our results revealed that the distribution across the studies exhibited an almost symmetrical trend, suggesting a low probability of publication bias (as depicted in **Supplementary Figure 1**).

## 4 Discussion

This study presents an all-encompassing summary of the available evidence regarding perioperative outcomes, renal function, and oncological outcomes connected with the utilization of LPN and OPN for addressing intricate renal tumors. Additionally, certain essential discoveries unearthed in this study underscore the requirement for further thorough exploration.

Laparoscopic surgery is employed in clinical practice; however, LPN demands the surgeon’s precise procedural expertise, encompassing tasks like tumor excision, reconstruction of the renal pelvis and calices, kidney hemostasis, and the intricate laparoscopic suture technique within confined spaces (22). As a result, OPN is favored for managing complex renal tumors due to its expansive surgical field of vision and broad spectrum of indications. The analysis indicated that LPN was associated with a longer operative time compared to OPN. This occurrence may be attributed to the following factors. Certain studies have employed Retroperitoneal LPN for complex renal tumors. The execution of retroperitoneal LPN necessitates meticulous surgical skills from the surgeon, encompassing tasks such as tumor excision, restoration of the renal pelvis, achieving kidney hemostasis, and executing precise suture techniques within confined spaces. Importantly, it should be noted that the learning curve for retroperitoneal LPN is extended and requires a heightened level of proficiency from the surgeon. Therefore, despite the array of advantages associated with retroperitoneal LPN, it continues to exhibit certain drawbacks, including an extended learning curve, elevated prerequisites for surgical proficiency among medical practitioners, and prolonged operative durations. Hence, further research is warranted to validate this conclusion. However, LPN demonstrated a shortened hospitalization period in comparison to OPN, primarily due to its reduced occurrence of postoperative complications, considering that complications can significantly prolong the duration of hospitalization (23). At the same time, the use of ultrasonic scalpel during operation can quickly remove the lesion, reduce intraoperative blood loss, and is more conducive to the recovery of intestinal function, thereby shortening the length of hospital stay (24, 25). However, the length of hospitalization is also influenced by institutional capacity and the proficiency of the surgeon. The rapid advancements in anesthesiology and the management of perioperative care might potentially contribute to a reduction in hospital stay. Thus, additional studies are imperative to validate this outcome.

Blood loss during surgery is a critical factor for assessing surgical quality. An important benefit of minimally invasive surgery is the reduction in operative blood loss. Our study revealed a noteworthy observation: LPN demonstrated reduced blood loss compared to OPN. This discrepancy can be attributed to the utilization of a laparoscopic vision imaging system, which provides an expansive surgical field of vision, enabling surgeons to promptly identify and address bleeding sites using electric coagulation forceps (26). These reasons could account for the lower blood loss observed in the LPN group compared to the OPN group. However, despite this finding, an analysis of cumulative outcomes indicated no statistically significant difference in transfusion rates between LPN and OPN. This phenomenon could be linked to the expertise of physicians and the established hospital transfusion protocols. However, a significant difference in the prevalence of transfusion rates between LPN and OPN was not observed. This lack of distinction could be attributed to the level of experience of the medical practitioners and the institution’s guidelines pertaining to blood transfusion, both of which have a potential impact on the transfusion rate.

The combined results suggested the absence of a statistically significant distinction in warm ischemia time between LPN and OPN. Moreover, certain elements deserve our focus. Some studies suggest that keeping warm ischemia time under 25 or 30 minutes is advisable to minimize the potential risk of detrimental effects on renal function (27, 28). In a recent study, Buffi et al. (29) presented the perioperative outcomes of 255 patients who underwent robot-assisted PN across four high-volume medical centers. The study revealed that 33.7% of patients experienced a warm ischemia time surpassing 20 minutes, while 7.8% had a warm ischemia time exceeding 30 minutes. In summary, the ischemic duration associated with LPN proves to be well-tolerated in the context of intricate renal tumors. Recent research has highlighted that warm ischemia time holds a relatively minor influence on long-term renal functional outcomes. However, preoperative renal function and the preservation of kidney count emerge as notably interconnected with the key determinants of long-term renal results (30). Furthermore, Fergany et al. (31) highlighted the significant role of age in the recovery of long-term renal function post-surgery. Thus, further research is essential to validate the influence of warm ischemia time on postoperative renal function. It is important to highlight that the results revealed no significant difference in the decline of estimated glomerular filtration rate (eGFR) between the LPN and OPN groups.

In terms of complications, we utilized the Clavien classification to assess surgical complications. LPN demonstrated a lower incidence of overall complications compared to OPN. For patients undergoing minimally invasive surgery, there is a notable reduction in both intraoperative blood loss and postoperative pain, which significantly benefits the recovery process. In the OPN group, the incision made between the 11th or 12th rib for partial nephrectomy is more invasive, exerting considerable stress on the body and increasing the likelihood of severe complications that can impede postoperative recovery (32). Kızılay et al. (33) emphasized that when comparing LPN with OPN for renal cancer treatment, they discovered noteworthy variations in postoperative C-reactive protein levels among patients undergoing LPN and OPN. This finding underscores that open surgery can trigger a heightened stress response in the body, potentially impacting postoperative recovery. Additionally, minimally invasive surgery offers several advantages, such as smaller incisions and limited anatomical exposure. These aspects contribute to a reduced risk of adjacent organ damage and, consequently, fewer postoperative complications.

The assessment of oncologic outcomes is of utmost importance. In fact, heightened tumor complexity appears to be linked with invasive traits and a lower survival rate. During partial nephrectomy, the presence of dense and adherent “sticky” perirenal fat surrounding the kidney can complicate the procedure and impede the precise demarcation of the surgical margin between the renal tumor and healthy tissue (34). This could potentially result in positive surgical margins and significant perioperative complications. However, A meta-analysis of PSM and OS revealed no statistically significant difference between LPN and OPN. However, there are several crucial considerations to be mindful of. Firstly, PSM might not necessarily serve as a definitive predictor of recurrence (35). Secondly, numerous factors could potentially influence the occurrence of PSM, including tumor diameter, surgical approach, and tumor stage (36). Consequently, additional research is imperative to validate the findings we have presented. Thirdly, due to a shortage of available literature, it is currently challenging to ascertain the potential differences in metastatic recurrence between the two groups, as well as their potential implications on recurrence-free survival.

Several other significant issues necessitate thorough discussion. The studies included in this analysis employed varying surgical methods for performing PN. Notably, the choice between retroperitoneal and intraperitoneal approaches introduced some heterogeneity across the studies. The retroperitoneal approach offers numerous advantages, including simplified ligation of the renal artery, thereby reducing blood loss during the isolation of renal tumors. Simultaneously, it minimizes interference with the intestines, subsequently lowering the risk of complications (37). However, it’s important to note that this approach does come with certain disadvantages, such as limited surgical space. Consequently, further investigations of high quality are imperative to ascertain the more suitable surgical approach for complex renal tumors. Secondly, the procedures encompassed within this study were executed by surgical teams possessing substantial expertise in laparoscopic surgery, and the data were sourced from large institutions. Moreover, it’s noteworthy that the selection of the surgical approach was not randomized; rather, it was guided by clinical and radiological evaluations. Consequently, the outcomes may not readily generalize to low-volume centers characterized by limited familiarity with laparoscopic techniques. Thus, further studies are imperative to substantiate our findings.

The study was conducted following the rigorous guidelines of PRISMA (13). Nonetheless, certain limitations influence the analysis. Firstly, all the studies were retrospective or prospective, ranging from low to moderate quality. Furthermore, no randomized controlled trials have been included in our study. Consequently, these studies are undoubtedly prone to inherent selection bias. Secondly, no subgroup analyses were conducted based on the surgical method (transperitoneal or retroperitoneal), potentially introducing some heterogeneity into the results. Furthermore, we conducted a pooled analysis of studies that employed kidney and PADUA scores ≥ 7, which might unveil subtle distinctions. However, the scarcity of adequate literature hindered our capacity to perform subgroup analyses. Finally, the included studies offered limited insights into outcomes in other oncology domains (e.g., free survival and cancer-specific survival), thus impeding a comparison of the two groups in terms of these outcomes.

## 5 Conclusions

LPN stands out as a more favorable choice than OPN for the management of intricate renal tumors. LPN brings forth benefits including a decreased hospitalization period, diminished blood loss, and a lower occurrence of complications. Moreover, LPN shows comparable oncological results when contrasted with OPN. However, it’s important to acknowledge that the majority of the included studies were of moderate quality and held a retrospective nature. Thus, additional research that integrates studies of superior quality and extended follow-up durations is imperative for a thorough comparison of the outcomes between these two approaches.

## Data availability statement

The original contributions presented in the study are included in the article/**Supplementary Material**. Further inquiries can be directed to the corresponding author.

## Author contributions

FZ: Conceptualization, Data curation, Formal analysis, Investigation, Methodology, Project administration, Resources, Software, Supervision, Validation, Visualization, Writing – original draft, Writing – review & editing. J-SH: Conceptualization, Data curation, Formal analysis, Investigation, Project administration, Software, Supervision, Validation, Visualization, Writing – original draft, Writing – review & editing. K-YZ: Conceptualization, Formal analysis, Investigation, Methodology, Project administration, Resources, Software, Supervision, Validation, Visualization, Writing – original draft, Writing – review & editing. X-HL: Conceptualization, Data curation, Formal analysis, Investigation, Methodology, Project administration, Resources, Visualization, Writing – original draft, Writing – review & editing.

## Supplementary material

Supplementary Figure 1 Funnel plot **(A)** operative time, **(B)** length of hospital stay, **(C)** warm ischemia time, **(D)** overall complication.
