# Supplementary figures and images for: Perioperative, functional, and oncologic outcomes of laparoscopic partial nephrectomy versus open partial nephrectomy for complex renal tumors: a systematic review and meta-analysis
Source: Front Oncol. 2024 Jan 10;13:1283935. doi: 10.3389/fonc.2023.1283935 (PMC10809712; doi:10.3389/fonc.2023.1283935)

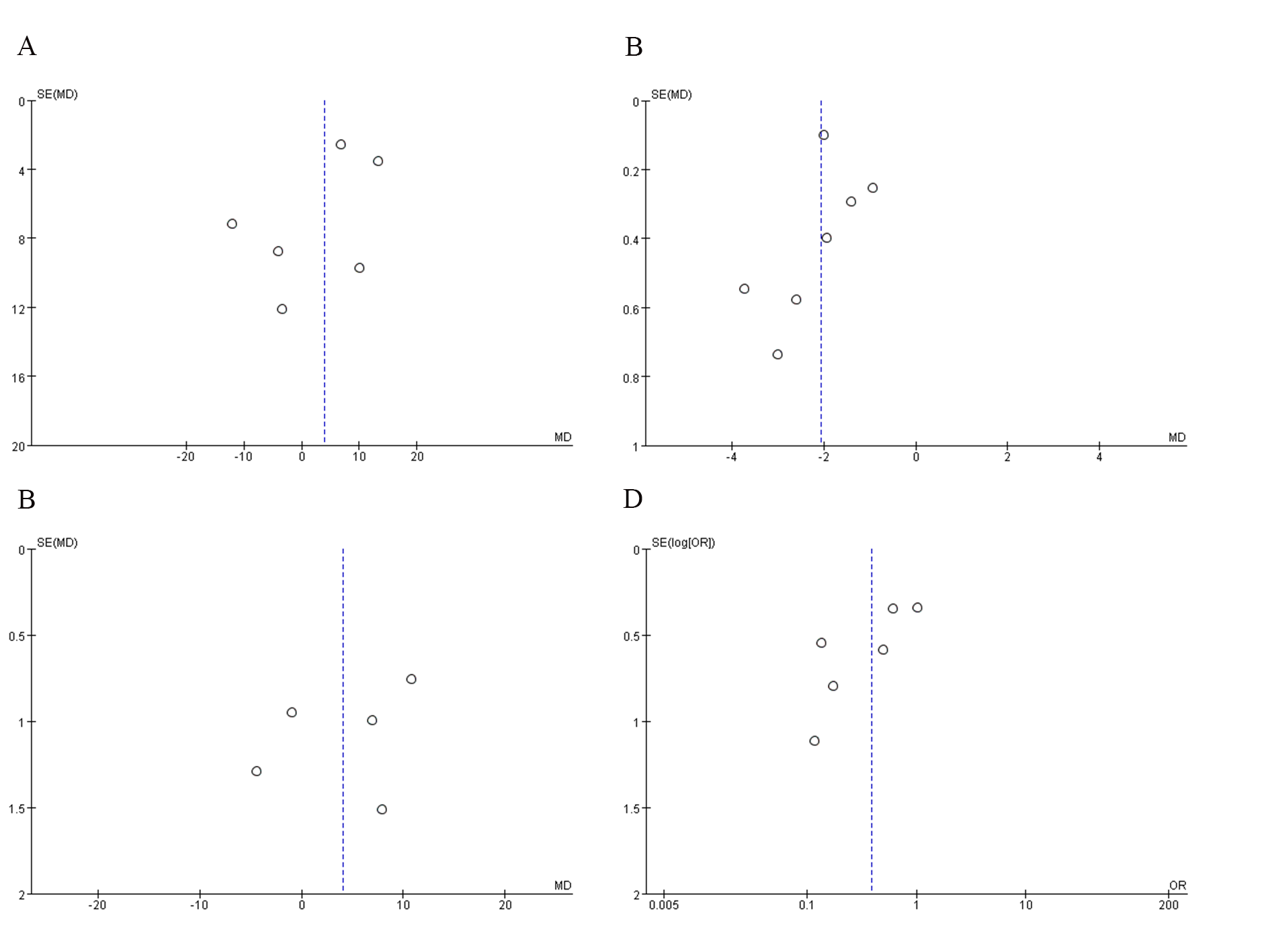

Supplement: Supplementary Figure 1 — Funnel plot (A) operative time, (B) length of hospital stay, (C) warm ischemia time, (D) overall complication. [file Image_1.tif]
