# Supplementary material for: Perioperative, functional, and oncologic outcomes of laparoscopic partial nephrectomy versus open partial nephrectomy for complex renal tumors: a systematic review and meta-analysis
Source: Front Oncol. 2024 Jan 10;13:1283935. doi: 10.3389/fonc.2023.1283935 (PMC10809712; doi:10.3389/fonc.2023.1283935)
Supplement: Supplementary file 2 [file Table_1.docx]

| **Table S1 The risk of bias(Non-RCTs)-ROBINS-I** | | | | | | | |  |
| --- | --- | --- | --- | --- | --- | --- | --- | --- |
| Bias domain | Giulioni | Liu | Guo | Li | Yu | Chiancone | Mari |  |
|  |  |  |  |  |  |  |  |  |
| Bias due to confounding | Moderate | Moderate | Moderate | Moderate | Moderate | Moderate | Moderate |  |
|  |  |  |  |  |  |  |  |  |
| Bias in selection of participants into the study | Low | Low | Moderate | Low | Moderate | Low | Low |  |
|  |  |  |  |  |  |  |  |  |
| Bias in classification of interventions | High | Moderate | Low | Moderate | Moderate | High | Moderate |  |
|  |  |  |  |  |  |  |  |  |
| Bias due to deviations from intended interventions | Low | Low | Moderate | Moderate | Low | Moderate | High |  |
|  |  |  |  |  |  |  |  |  |
| Bias due to missing data | Moderate | Moderate | Moderate | Low | Moderate | Low | Low |  |
|  |  |  |  |  |  |  |  |  |
| Bias in measurement of outcomes | Moderate | Moderate | Low | High | Low | Moderate | Moderate |  |
|  |  |  |  |  |  |  |  |  |
| Bias in selection of the reported result | Moderate | Low | Moderate | Moderate | Moderate | Moderate | Moderate |  |
|  |  |  |  |  |  |  |  |  |
| Overall bias | High | Moderate | Moderate | High | Moderate | High | High |  |
|  |  |  |  |  |  |  |  |  |
