# Supplementary material for: Perioperative, functional, and oncologic outcomes of laparoscopic partial nephrectomy versus open partial nephrectomy for complex renal tumors: a systematic review and meta-analysis
Source: Front Oncol. 2024 Jan 10;13:1283935. doi: 10.3389/fonc.2023.1283935 (PMC10809712; doi:10.3389/fonc.2023.1283935)
Supplement: Supplementary file 3 [file Table_2.docx]

| **Table S2 Sensitivity analysis** | | |  |
| --- | --- | --- | --- |
|  |  |  |  |
| Outcomes | Excluding study | Heterogeneity I^2^ (%) |  |
|  |  |  |  |
| Hospital stay | Giulioni | 83 |  |
|  |  |  |  |
|  | Liu | 84 |  |
|  |  |  |  |
|  | Guo | 85 |  |
|  |  |  |  |
|  | Li | 84 |  |
|  |  |  |  |
|  | Yu | 77 |  |
|  |  |  |  |
|  | Chiancone | 71 |  |
|  |  |  |  |
|  | Mari | 83 |  |
|  |  |  |  |
| Blood loss | Giulioni | 91 |  |
|  |  |  |  |
|  | Liu | 92 |  |
|  |  |  |  |
|  | Guo | 90 |  |
|  |  |  |  |
|  | Li | 82 |  |
|  |  |  |  |
|  | Yu | 86 |  |
|  |  |  |  |
|  | Chiancone | 92 |  |
|  |  |  |  |
|  | Mari | 92 |  |
|  |  |  |  |
| Warm ischemia time | Giulioni | 97 |  |
|  |  |  |  |
|  | Liu | 96 |  |
|  |  |  |  |
|  | Guo | 97 |  |
|  |  |  |  |
|  | Li | 98 |  |
|  |  |  |  |
|  | Yu | 98 |  |
|  |  |  |  |
|  | Mari | 97 |  |
|  |  |  |  |
| eGFR decline | Giulioni | 96 |  |
|  |  |  |  |
|  | Guo | 95 |  |
|  |  |  |  |
|  | Yu | 96 |  |
|  |  |  |  |
|  | Mari | 96 |  |
|  |  |  |  |
